# Insights into the roles of sperm in animal cloning

**DOI:** 10.1186/s13287-020-01599-6

**Published:** 2020-02-18

**Authors:** Pengxiang Qu, Yongsheng Wang, Chengsheng Zhang, Enqi Liu

**Affiliations:** 1 Laboratory Animal Center, Xi’an Jiaotong University Health Science Center, No.76, Yanta West Road, Xi’an, 710061 Shaanxi China; 2 Key Laboratory of Animal Biotechnology of the Ministry of Agriculture, College of Veterinary Medicine, Northwest A&F University, Yangling, 712100 Shaanxi China; 3 Precision Medicine Center, The First Affiliated Hospital of Xi’an Jiaotong University, Xi’an, 710061 China; 4 The Jackson Laboratory for Genomic Medicine, Farmington, CT 06032 USA

**Keywords:** Cytoskeleton remodeling, Nucleus reprogramming, Sperm, Somatic cell nuclear transfer, Animal cloning

## Abstract

Somatic cell nuclear transfer (SCNT) has shown a wide application in the generation of transgenic animals, protection of endangered animals, and therapeutic cloning. However, the efficiency of SCNT remains very low due to some poorly characterized key factors. Compared with fertilized embryos, somatic donor cells lack some important components of sperm, such as sperm small noncoding RNA (sncRNA) and proteins. Loss of these factors is considered an important reason for the abnormal development of SCNT embryo. This study focused on recent advances of SCNT and the roles of sperm in development. Sperm-derived factors play an important role in nucleus reprogramming and cytoskeleton remodeling during SCNT embryo development. Hence, considering the role of sperm may provide a new strategy for improving cloning efficiency.

## Introduction

Somatic cell nuclear transfer (SCNT) has shown great advantages and application prospects in the generation of transgenic animals, protection of endangered animals, and stem cell therapy [1]. With the rapid development of gene-editing biotechnology, the combination of gene editing and SCNT has produced more than 300 genetically modified animals, including pigs, cattle, sheep, and goats. It has great potential in the genetic engineering of livestock [2]. Also, SCNT is probably one of the ways to save endangered species, but studies on the use of SCNT (both intra- and interspecies SCNT) in wildlife species are scarce [3]. The utmost relevance of SCNT in regenerative medicine remains unquestionable due to the fact that it is not only free of somatic epigenetic memory but also more similar to conventional embryonic stem cells (ESCs) derived from in vitro fertilized embryos in their transcriptomic and epigenomic signatures [4]. In SCNT, a somatic cell nucleus from the patient is injected into an enucleated oocyte, and the resulting ESCs are then isolated from cloned blastocysts. These ESCs are capable of differentiating into all cell types, which is a potential approach to treat Parkinson’s disease, lateral amyotrophic sclerosis, and other disorders [5]. However, the use of this technology in clinical treatment may take a long time due to its low efficiency, unknown mechanisms, and other ethical issues.

Since the birth of the first cloning sheep “Dolly” in 1996, various species have been successfully cloned [6–23] (Table 1). Although SCNT has shown great progress over the past two decades, the breakthrough is primarily in different species, and the efficiency is still very low [24]. Common concerns include inferior developmental competence of SCNT embryos, low pregnancy rate, fetal placental abnormalities, large offspring syndrome, immunodeficiency, and early death in the pups [25]. Still many aspects of SCNT remain unknown, and more in-depth studies are urgently needed. Currently, the reasons for the low efficiency of SCNT are roughly classified into the following two aspects: (1) Operations, such as enucleation, electrofusion, and embryo culture, can cause endoplasmic reticulum stress and damage. Changes in the osmotic pressure and accumulation of toxic metabolites in the culture medium can lead to SCNT embryo apoptosis [26]. (2) SCNT embryos manifest abnormal epigenetic and expression patterns, which are currently considered to be the main barrier for reprogramming [24].

**Table 1:** Summary of the cloned mammalian species

Species	Published year	Donor cell type	Born number	Transferred embryos number	Reference
Sheep	1996	Epithelium	1	29	[6]
		Fibroblast	2	34	
Bovine	1998	Fetal fibroblast	3	28	[7]
Mouse	1998	Cumulus cell	31	1385	[8]
		Sertoli cell	1	59	
		Neuron	1	46	
Goats	1999	Fetal fibroblast	1	47	[9]
Pig	2000	Granulosa cell	5	72	[10]
Mouflon	2001	Granulosa cell	1	7	[11]
Rabbit	2002	Cumulus cell	6	371	[12]
Cat	2002	Fibroblast	1	81	[13]
		Cumulus cell			
Horse	2003	Fibroblast	1	17	[14]
Mule	2003	Fibroblast	3	305	[15]
Rat	2003	Fibroblast	3	129	[16]
Dog	2005	Fibroblast	2	1095	[17]
Ferrets	2006	Fibroblast	1	104	[18]
		Cumulus cell	3	193	
Wolf	2007	Fibroblast	2	251	[20]
Buffalos	2007	Granulosa cell	3	42	[21]
		Fibroblast			
Red deer	2007	Periosteum	8	84	[19]
		Bone cell			
		Fat cell			
Camel	2010	Cumulus cells	1	139	[22]
		Fibroblast			
Monkey	2018	Fibroblast	2	79	[23]

### Abnormal nuclear reprogramming of SCNT embryos

The DNA sequence of most cells in an individual is exactly the same, but the gene expression patterns in different cell types vary widely. This disparity, which is also regulated by epigenetics, leads to diverse morphology and functions. Histone modification, DNA methylation, genomic imprinting, and noncoding RNA regulation are the main forms of epigenetics. The original epigenetic modifications in donor cell genome are incompletely erased and abnormally reprogramed by the enucleated oocyte cytoplasm.

### Histone modification

In fertilized embryos, protamine-encapsulated DNA in sperm chromatins are exchanged by maternal histones, while donor cell histones are rapidly replaced by maternal histones in SCNT embryos [27]. Histone modification plays an important role in regulating gene expression of the reprogramming process. The level of histone acetylation in SCNT embryos is low, and histone deacetylase inhibitors (HDACis), such as trichostatin A (TSA), Scriptaid, and Oxamflatin, are used to increase the level of histone acetylation, significantly improving the SCNT efficiency in mice, bovine, pigs, monkeys, and other mammals [23]. Similar to the adjustment of histone acetylation level, an abnormally high level of H3K9me3 modifications in the heterochromatin region of SCNT embryos can be adjusted by the overexpression of demethylase (KDM4A or KDM4D) or downregulation of methyltransferase (Suv39h1 and Suv39h2) [28]. H3K27me3 is also considered an epigenetic barrier for reprogramming because of its association with the loss of imprinting. The amelioration of abnormal H3K27me3 and H3K4me3 levels in SCNT embryos is another mechanism to improve SCNT efficiency [29].

### DNA methylation

Methylation at the 5-position of cytosine (5mC) is an important form of epigenetic modification during mammalian embryo development, which is established and maintained by DNA methyltransferase (Dnmts) and demethylated ten-eleven translocation (TET) protein. DNA methylation of the donor cell genome is a relatively stable marker, and the level of 5mC is significantly higher in SCNT embryos than in fertilized embryos during preimplantation stage [28]. The unusual DNA methylation pattern mostly exists on the satellite sequence, long terminal repeat sequence, long dispersed elements (LINEs), and imprinted control region (ICR). Trophoblast cells in SCNT blastocysts are hypermethylated in intergenic differentially methylated regions, which are associated with subsequent abnormal placental development [30]. Treatment with a methyltransferase inhibitor and interference of Dnmts have been used to reduce the higher DNA methylation level, and this strategy effectively improves the SCNT efficiency [28].

### Imprinting

Maternal and paternal genomes contain indispensable components for both embryonic development, and they also play complementary roles in development. Within imprinted genes, only one working copy exists because either maternal copy or paternal copy is silenced. In mammals, most of the epigenetic tags are reprogramed after fertilization, while only imprinted genes keep their epigenetic tags [31]. Improper imprinting can result in two active copies or two inactive copies, leading to severe developmental abnormalities, cancer, and other problems [32]. During the process of injecting a donor nucleus from a nonreproductive cell into an enucleated oocyte in SCNT, imprinting of sperm and oocyte does not exist. Loss of H3K27me3 imprinting in SCNT embryos disrupts post-implantation development [33]. A large number of studies provided the evidence of the abnormal expression of imprinted genes in SCNT animal tissues with abnormalities. Compared with normal reproductive cattle, H19 was highly expressed in the bladder, brain, heart, and lung of dead cloned cattle. In the meantime, IGF2 was highly expressed in the heart, kidney, lung, and spleen, and IGF2R was highly expressed in the bladder and brain of dead cloned cattle [34]. The abnormal expression of imprinted genes was also found in pig, mouse, and other cloned animals [35, 36]. Ameliorating the abnormal imprinting pattern of SCNT embryo might be a promising strategy to improve the SCNT efficiency.

### X-chromosome inactivation

During mammalian development, X chromosome inactivation (XCI) is observed in the preimplantation stage of embryos and is inherited to the placental lineage to balance the gene expression on the X chromosome. XCI is triggered by Xist, which is both an imprinted gene and a noncoding RNA [37]. Xist expression was abnormally high in male or female SCNT-derived mouse embryos, leading to the inactivation of almost all X chromosomes [38]. Deletion or downregulation of Xist resulted in a tenfold increase in the birth rates of cloned offspring in mice [38]. Also, other studies have corroborated that the rectification of Xist in preimplantation SCNT embryos has long-term effects on their development and postnatal growth.

### Abnormal cytoskeleton remodeling of SCNT embryo

Significant differences in cytoskeletal organization and post-transcriptional modification of cytoskeleton were observed in SCNT and IVF embryos. The abnormal cytoskeleton remodeling was considered to be responsible for the abnormal development of SCNT embryos, in particular abnormal pronuclear structures, faster development kinetics, and higher ratio of blastomere fragments [39].

### Cytoskeleton remodeling

The cytoskeleton is composed mainly of microtubules, intermediate filaments (IFs), and microfilaments. The functions of cytoskeleton include regulating intracellular material transport, organelle positioning, maintaining cell shape, regulating cell movement and cell division, and maintaining genomic stability. Vimentin is a type III intermediate filament expressed in fibroblasts, leukocytes, and blood vessel endothelial cells. Studies have found that vimentin persists around the nucleus and damages the DNA of SCNT embryos [40]. Studies on rhesus monkeys have shown that the nuclear mitotic protein (NUMA) cannot recombine in SCNT embryos, causing aneuploidy [41]. The spindle–chromosome complex (SCC) in SCNT embryos showed a higher abnormality rate [42]. Removal of the oocyte SCC can cause an irreversible loss or damage of some cytoplasmic proteins, leading to mitotic abnormalities and aneuploidy in early SCNT embryos.

After a donor cell nucleus is injected into the enucleated oocyte, the M phase–promoting factor (MPF) in oocyte triggers nuclear envelope breakdown (NEBD) and premature chromosome condensation (PCC). In normal fertilized eggs, the sperm-derived protein PLCZ1 activates zygotes by Ca^2+^ oscillation and decomposition of MPF. Due to the lack of activating factors in somatic cells, artificial activation is needed in SCNT embryos to initiate development. In fertilized eggs, the two nuclei from sperm and oocytes are known as male pronucleus and female pronucleus, while the pronucleus in the SCNT embryo is called pseudo-pronucleus. Abnormality can often be observed in the formation of NEBD, PCC, and pseudo-nucleus of SCNT embryos during the first cell cycle [43]. Abnormal centrosomes and failed “pronuclear” migration are related to errors in spindle morphology, chromosome alignment, and cytokinesis in SCNT embryos, which can lead to chromosome instability, disorder of transcriptional regulation and DNA replication, and DNA damage [44]. The complete deletion of sperm regulators in SCNT embryos may be an important reason for the abnormal PCC and pronucleus formation [39].

### Post-translational modification (PTM) of cytoskeleton

There are many forms of PTM, such as phosphorylation, glycosylation, ubiquitination, tyrosinylation, glutamination, glycosylation, acetylation, and SUMOylation. These modifications enable cytoskeletal proteins to perform multiple functions in different organelles and physiological states. Acetylation of lysine at the end of α-tubulin is crucial to cytoskeletal stability [45]. Tyrosinization of microtubules regulates the separation of chromosomes in the late stage of cell division via monitoring the activity of depolymerization kinesin [46]. Polyglutamate modification can induce microtubule shear, regulate the length of mitotic spindles, and control the timely division of cytoplasm. Phosphorylation occurs most frequently on serine, threonine, and tyrosine residues of the cytoskeleton. It participates in various cell cycle pathways [47]. Glycosylation of cytoskeleton involves protein folding, distribution, stability, and activity [48]. SUMOylation of cytoskeleton is involved in cell cycle regulation and DNA damage responses.

PTM of cytoskeleton plays a vital role in fertilization, symmetric and asymmetric cell divisions, morula and blastocyst formation, stem cell maintenance, and differentiation. Particularly in the period before the zygotic genome activation (ZGA) stage, the embryonic genes are silenced, and PTM-mediated cytoskeletal remodeling dramatically changes, which is a rapid and efficient way to regulate embryonic developmental events [49]. PTM mediation by sperm was thought to be critically important for cytoskeletal remodeling, but the knowledge of these events is still sparse. α-Tubulin in microtubules of mouse embryos is acetylated in a specific spatial and temporal sequence during the preimplantation of embryo development [50]. Tyrosinated α-tubulin redistributes toward the apex of cells during the 8-cell and 16-cell stages, while acetylated α-tubulin accumulates near the intercellular contact zone in the basal part of the cell [49]. During the morula stage, acetylated α-tubulin is detected only in a subpopulation located predominantly at the cell cortices [49]. During the blastocyst stage, the cells inside the developing embryos contain more acetylated microtubules compared with the outside cells [49]. The drastic PTM-mediated cytoskeletal remodeling undoubtedly plays an important role during embryo development. A recent study found a different α-tubulin acetylation pattern between SCNT and fertilized embryos [39].

### Roles of sperm in reprogramming

**Fig. 1:**
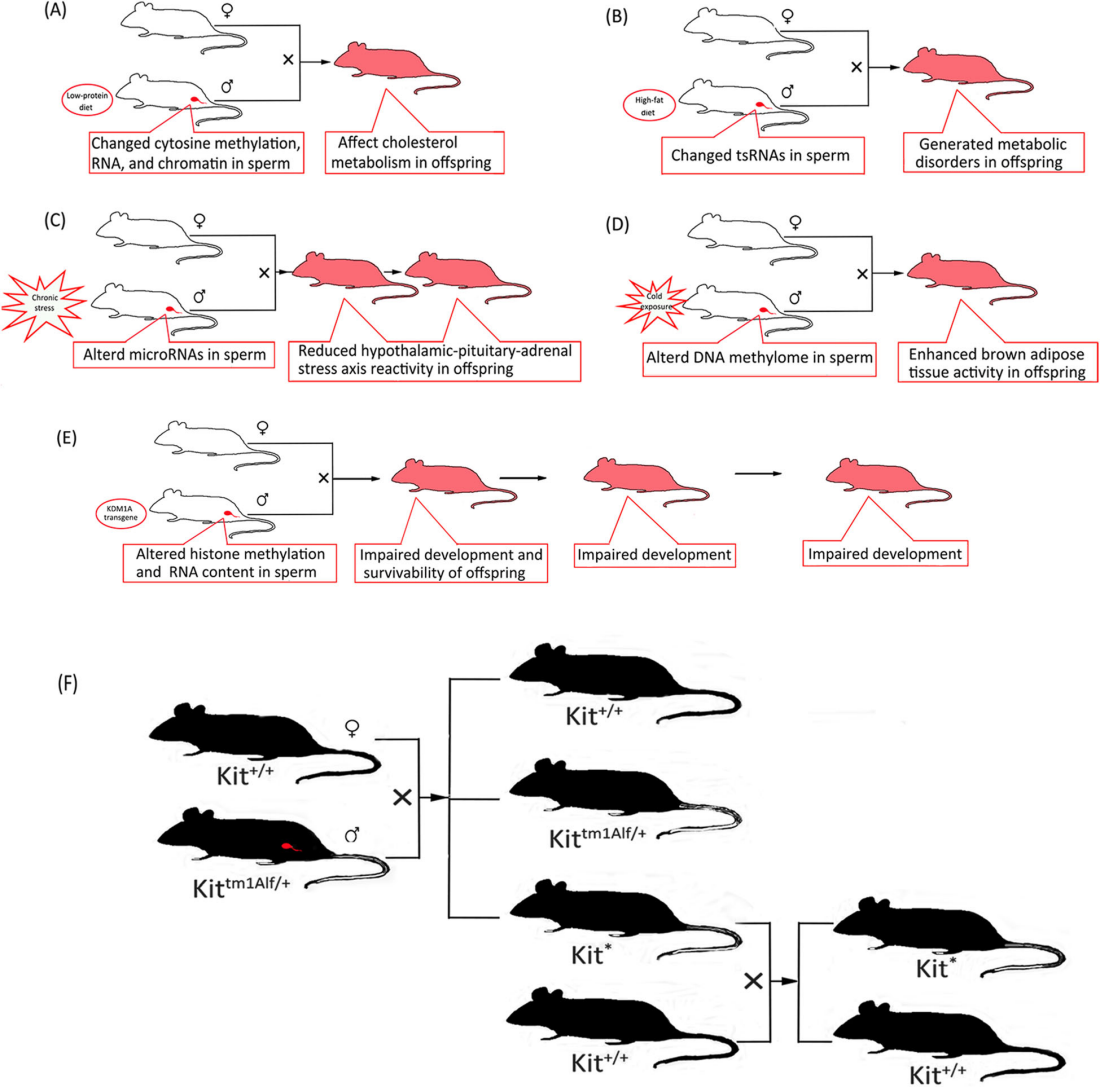
Intergenerational and transgenerational epigenetic inheritance via the sperms in mice. **a** Cytosine methylation, RNA, and chromatin of sperm in male fed a low-protein diet changed, and the offspring exhibited elevated hepatic expression of many genes involved in lipid and cholesterol biosynthesis and decreased levels of cholesterol esters. **b** Sperm tsRNAs in male fed a high-fat diet exhibited changes in expression profiles and RNA modifications, and this treatment generated metabolic disorders in the F1 offspring. **c** Chronic paternal stress altered the profiling of sperm microRNAs, which reduced hypothalamic–pituitary–adrenal (HPA) stress axis reactivity in the offspring. **d** Seasonal or experimental cold exposure induced the epigenetic programming of the sperm such that the offspring harbored hyperactive brown adipose tissue and an improved adaptation to overnutrition and hypothermia. **e** Disruption of histone methylation in developing sperm by exposure to the KDM1A transgene severely impaired development and survivability of offspring, and the defects occurred in nontransgenic descendants in the absence of KDM1A germline expression. **f** The genotype of male heterozygous mice Kit^tm1Alf/+^ showed a white tail tip and white feet, and the genotype of mice Kit^+/+^ showed a full color. The male mice Kit^tm1Alf/+^ were mated with the female mice Kit^+/+^, and the offspring were of three kinds: offspring (Kit^+/+^) showed a full color, offspring (Kit^tm1Alf/+^) showed a white tail tip and white feet, and offspring (Kit^*^) showed a white tail tip and white feet. The genotype of Kit^+/+^ and Kit^*^ was the same, but the phenotype was different because Kit messenger RNA decreased in the offspring (Kit^*^). Male mice (Kit^*^) were mated with female mice (Kit^+/+^), the offspring still had a white tail tip and white feet, and the degree of this phenomenon reduced

During the process of fertilization, the paternal and maternal genomes undergo genome-wide epigenetic modifications or reprogramming and transform into an early embryonic genome model with developmental pluripotency, thus starting a new life course. This process is the period during which the epigenetic modification of the genome is the most frequent and the development events are most concentrated. It is traditionally believed that oocyte modulators are the main factors affecting epigenetic reprogramming and embryonic development, while the sperm contributes only to paternal chromosomes and activates fertilized embryos. The latest studies showed that sperm proteins [51], protein modification [52, 53], DNA methylation [54, 55], and sperm small noncoding RNA (sncRNA) [56–58] were all important carriers for regulating epigenetics. These epigenetic factors can escape the reprogramming of early embryos and regulate genes at the transcriptional, post-transcriptional, translational, or post-translational levels, thus contributing to the embryo, and even offspring, development (Fig. 1), [51–58]. It is also worth mentioning that seminal plasma has an important role on offspring development and health [59, 60].

### Sperm sncRNA

**Fig. 2:**
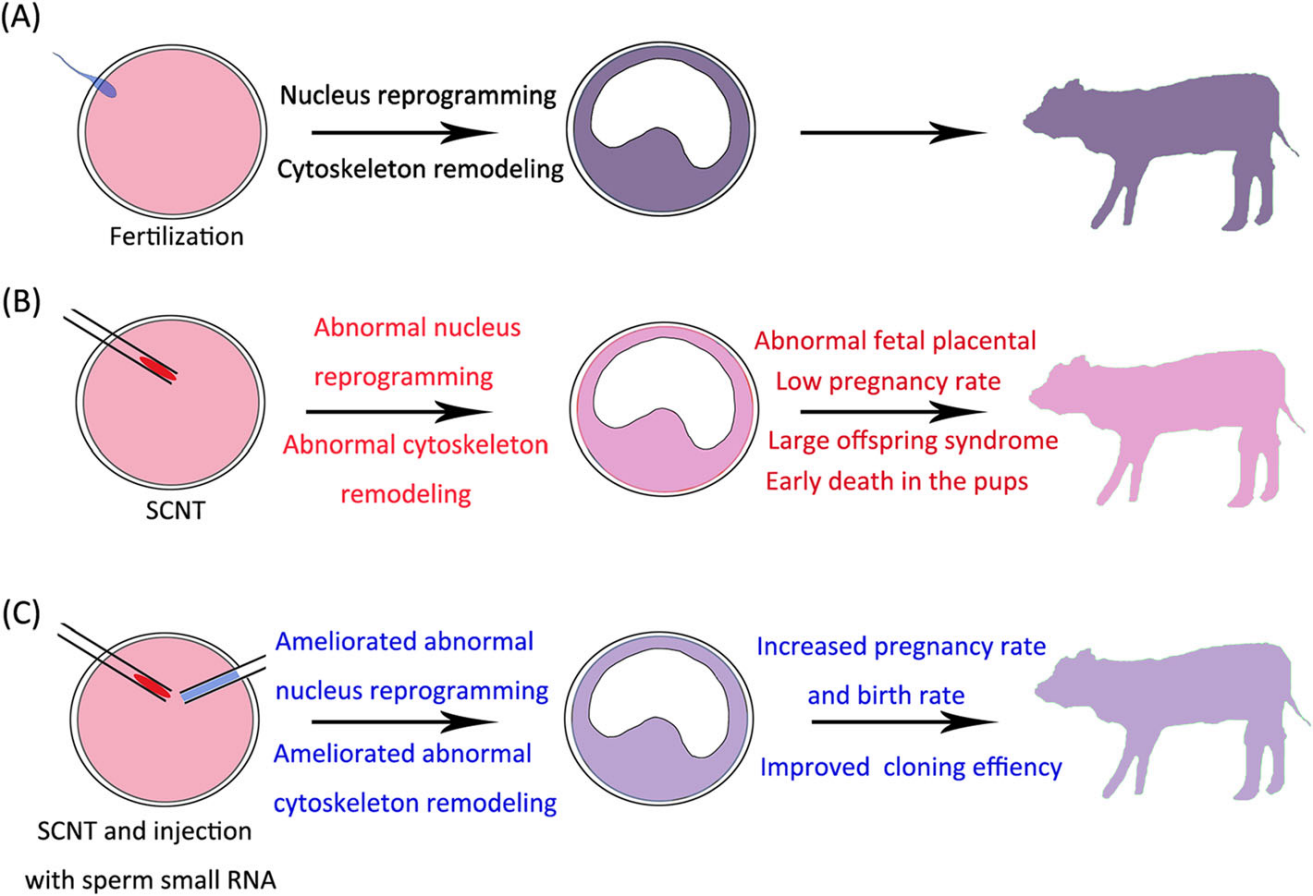
Diagram showing the development of fertilized and SCNT embryos; SCNT embryos had sperm small RNA in cattle. **a** Sperm enters oocytes, fertilizes, and initiates nucleus reprogramming and cytoskeleton remodeling. **b** Somatic cells were injected into enucleated oocytes, and SCNT embryos manifest abnormal nucleus reprogramming and cytoskeleton remodeling. **c** Sperm small RNAs were injected into SCNT embryos, and the treatment ameliorated abnormal nucleus reprogramming and cytoskeleton remodeling in SCNT embryos in cattle

Sperm contains both coding and noncoding RNAs, including mRNA, miRNA, piRNA, endo-siRNA, and tRNA. Sperm-borne RNA plays an important role in embryonic development following fertilization. Sperm small RNA could regulate the epigenetic state and cytoskeleton remodeling in SCNT embryos (Fig. 2), [39]. Many studies have been performed on individual sample types, such as oocytes, sperm, or donor cells. However, a few studies included all three cell types. In the study by Yang et al., the deep sequencing method was used to measure the expression profiles of sncRNA in sperm, oocytes, and embryos. They used the expression profiles of CD4 + T cell (CD4) as a somatic cell control for illustrating the categories and length distributions of small RNAs [61]. In this review, the expression data of sperm, oocytes, and CD4 in a study by Yang et al. were selected to investigate the potential function of microRNA in sperm. A total of 21 microRNAs were highly expressed in sperm, and 65 microRNAs were only expressed in sperm. The microRNAs only expressed or highly expressed in sperm were compared with conservative microRNA among four species (mice, rats, humans, and rabbits) screened by Yuan et al. [62]. Nine microRNAs (miR-34c-3p, miR-99a-5p, miR-10b-5p, miR-135a-5p, miR-34c-5p, miR-200b-3p, miR-125b-5p, miR-34b-3p, and miR-199a-3p) were found in both sets. Target genes of these nine microRNAs were predicted using microDB (http://mirdb.org/mining.html). Then GO analysis and KEGG pathway analysis were conducted (Table 2). The top enriched biological processes of target genes were related to the regulation of transcription, indicating that sperm microRNAs might have an important role in the regulation of embryonic transcription. The enriched cellular component comprised mainly of cytoplasm, membrane, and nucleus, and the enriched molecular function included protein binding, metal ion binding, DNA binding, nucleotide binding, and transferase activity. These target genes were involved in important signaling pathways related to embryonic development, such as MAPK, cAMP, AMPK, regulation of actin cytoskeleton, and regulating pluripotency of stem cells.

**Table 2:** The five most-enriched GO categories and KEGG pathways for the target genes of conserved and high sperm miRNAs (*P* < 0.05, FDR < 0.05)

ID	Term	Count	*P* value	FDR
Biological process				
GO:0006351	Transcription, DNA-templated	254	2.65E−20	4.99E−17
GO:0045944	Positive regulation of transcription from RNA polymerase II promoter	155	7.38E−18	1.39E−14
GO:0006355	Regulation of transcription, DNA-templated	283	1.17E−17	2.21E−14
GO:0045893	Positive regulation of transcription, DNA-templated	95	1.89E−12	3.56E−09
GO:0000122	Negative regulation of transcription from RNA polymerase II promoter	111	4.19E−12	7.88E−09
Cellular component				
GO:0005737	Cytoplasm	668	1.71E−22	2.56E−19
GO:0016020	Membrane	634	4.87E−10	7.31E−07
GO:0005634	Nucleus	588	1.92E−15	2.83E−12
GO:0005654	Nucleoplasm	208	4.08E−08	6.12E−05
GO:0005829	Cytosol	194	5.85E−08	8.78E−05
Molecular function				
GO:0005515	Protein binding	473	8.29E−24	1.34E−20
GO:0046872	Metal ion binding	353	9.70E−11	1.57E−07
GO:0003677	DNA binding	227	5.16E−13	8.37E−10
GO:0000166	Nucleotide binding	198	2.71E−05	0.043909
GO:0016740	Transferase activity	161	4.83E−06	0.007827
KEGG pathway				
mmu04151	PI3K-Akt signaling pathway	58	3.05E−08	3.97E-05
mmu04010	MAPK signaling pathway	46	4.87E−08	6.34E−05
mmu04810	Regulation of actin cytoskeleton	38	1.74E−06	0.002264
mmu04152	AMPK signaling pathway	27	3.35E−06	0.004361
mmu04550	Signaling pathways regulating pluripotency of stem cells	27	1.40E−05	0.018295

The expression profiles of microRNA were different between sperms with a high fertilization rate and those with a low fertilization rate. Germline-specific Dicer and Drosha conditional knockout mice produced sperm partially deficient in microRNA, which disrupted maternal transcript turnover and failure in ZGA and caused a significant reduction in developmental potential. The findings in recent years showed that sperm-borne microRNA-449b improved epigenetic reprograming and decreased the apoptosis index of early embryos [63]. Also, microRNA-125b was a key epigenetic regulatory factor that promoted nuclear reprogramming via targeting SUV39H1, which played a vital role in heterochromatin organization, chromosome segregation, and mitotic progression [64]. In another study, deep sequencing was used to analyze small RNA in bovine sperm, and microRNA-125b was found to be highly expressed in bovine sperm [65]. Also, the target genes of sperm microRNAs were associated with placental development, and these imprinted genes were partially responsible for abnormal placenta development and large offspring syndrome [66].

piRNA plays an important role in mammalian spermatogenesis. Impairment of Miwi protein, which binds to piRNA, can lead to a higher exchange barrier between histones and protamines, which severely affect the stability of genomic DNA and chromatin remodeling, leading to male sterility. BTBD18, produced by the meiosis of mouse spermatogenic cells, regulates piRNA via promoting the transcriptional elongation of RNA polymerase II. By silencing BTBD18, the piRNA synthesis and spermatogenesis are disordered, resulting in male mouse sterility. piRNA is also involved in maternal mRNA degradation and plays an important role in regulating protein translation, mediating ubiquitination degradation, germ cell development, sex determination, and embryonic development. The latest study found that H3K9me3 in SCNT embryos significantly reduced, and injecting small RNA into SCNT embryos could significantly enhance cloning efficiency [39]. The molecular mechanism of action of sperm small RNA in regulating SCNT embryo reprogramming is still not clear and needs to be explored in subsequent studies.

### Sperm proteins

Up to date, a large number of proteomic studies on sperm or oocytes have been performed. Of the total 6871 proteins found in human mature sperm, 560 have been found to be involved in modulating gene expression, DNA methylation, histone modifications, and noncoding RNA biogenesis, which may be critical for regulation after ZGA and epigenetic transmission of altered phenotypes [51]. It is traditionally considered that zygote is affected mainly by proteins accumulated by oocytes before ZGA, while after ZGA, the embryo is regulated mainly by the embryonic proteins. In fact, sperm enters the oocyte carrying a large amount of proteins, and the half-life of these proteins can vary from 30 min to 8 days, indicating that some sperm proteins may play a role in the development of preimplantation stage embryos [67]. Sperm proteins are necessary for fertilization and embryo development. Sperm protein IZUMO1 guides egg–sperm fusion by binding to oocyte protein JNUO [68]. Sperm protein PLCZ1 induces Ca^2+^ oscillation, initiates pronuclear formation, and contributes to embryogenesis. Deficiency or abnormal positioning of sperm protein EQTN can lead to abnormal fertilization and cause disorders of gamete fusion [69]. Knockout of some sperm proteins can cause abnormal development at the preimplantation stage, death, or sterility.

### Roles of sperm in cytoskeleton remodeling

Recent studies on the regulation of early embryo development focused mainly on epigenetic modification, while a few studies investigated the role of cytoskeleton PTMs. In the last decade, efforts were made to improve the efficiency of SCNT transgenic animals with some achievements. Previous studies found that SCNT embryos showed higher abnormal cytoskeleton remodeling, such as multiple pronuclei formation and abnormal cleavage rate or time. They also demonstrated that sperm microRNAs or proteins derived from sperm were involved in cytoskeletal remodeling. Injecting sperm miR-449b into SCNT embryos slowed down the first cleavage time and improved the development potential of SCNT embryos [63]. Injecting bovine sperm small RNA into SCNT embryos ameliorated the acetylation of α-tubulin K40 and pronuclei formation rate, demonstrating that sperm small RNA played an important role in the cytoskeletal remodeling of SCNT embryos [39]. Proteins, which are rich in sperm, are the most direct regulators, and some proteins are important enzymes for embryonic development. Protamine 1 is an important protein in mature sperm, which binds with high affinity to all DNAs. Upon fertilization, paternal chromosomes rapidly lose protamine 1 and regain a nucleosomal organization built on maternal histones. Establishing somatic donor cells, which exogenously expressed protamine 1, could create spermatid-like chromatin and cytoskeleton structures [70]. The spermatid-like somatic cells were conducive to nuclear reprogramming and beneficial for SCNT [70]. Despite all this, a few studies have examined sperm proteins on SCNT embryos, and attempts are being made to study sperm-derived key proteins associated with SCNT development. Also, an in-depth investigation of cytoskeletal protein modification may be important for understanding embryo development networks and improving SCNT efficiency.

## Conclusion and future perspectives

Factors derived from sperm play important roles in nuclear reprogramming and cytoskeletal remodeling during embryo development. Loss of sperm-derived regulators in somatic cells may be an important reason for low cloning efficiency. The roles of sperm factors in epigenetic inheritance and fertilized embryo development increasingly attracted researchers’ attention. A few reports are available on the influence of sperm in SCNT embryos and additional studies on the involvement of sperm factors in embryo development are warranted. Sperm-specific and crucial RNA, proteins, and/or other epigenetic factors should be screened, and a regulatory network of these sperm factors on embryo development should be constructed. Furthermore, the involvement of sperm in nuclear reprogramming and cytoskeletal remodeling in fertilized and SCNT embryos should be elucidated. Considering the roles of sperm factors may improve the cloning efficiency and open a new era for SCNT.

## Data Availability

Not applicable.

## Abbreviations

SCNT: Somatic cell nuclear transfer
sncRNA: Small noncoding RNA
ESCs: Embryonic stem cells
HDACis: Histone deacetylase inhibitors
TSA: Trichostatin A
KDM4A: Lysine demethylase 4A
KDM4D: Lysine demethylase 4D
Suv39h1: Suppressor of variegation 3–9 homolog 1
Suv39h2: Suppressor of variegation 3–9 homolog 2
H3K27me3: Histone H3 lysine 27 trimethylation
H3K4me3: Histone H3 lysine 4 trimethylation
5mC: 5-Position of cytosine
Dnmts: DNA methyltransferase
TET: Ten-eleven translocation
LINEs: Long dispersed elements
ICR: Imprinted control region
H19: H19 imprinted maternally expressed transcript
IGF2: Insulin-like growth factor 2
IGF2R: Insulin-like growth factor 2 receptor
XCI: X chromosome inactivation
IVF: In vitro fertilization
IFs: Intermediate filaments
NUMA: Nuclear mitotic protein
SCC: Spindle–chromosome complex
MPF: M phase–promoting factor
NEBD: Nuclear envelope breakdown
PCC: Premature chromosome condensation
PLCZ1: Sperm protein PLCzeta
PTM: Post-translational modification
ZGA: Zygotic genome activation
CD4: CD4 + T cell
MAPK: Mitogen-activated protein kinase
cAMP: Cyclic adenosine monophosphate
AMPK: Adenosine 5′-monophosphate activated protein kinase
BTBD18: BTB domain containing 18
IZUMO1: Izumo sperm-egg fusion 1
JNUO: IZUMO1 receptor
EQTN: Equatorin
